# Kentucky Medical Society

**Published:** 1854-03

**Authors:** 


### KENTUCKY MEDICAL SOCIETY.

The State Medical Society of Kentucky was organized, at Frankfort, in October 1851. The following October it met at Louisville, and was well attended. The proceedings excited a great deal of interest. A number of very able reports were read, and formed a volume of transactions creditable to the medical profession of Kentucky. The Third meeting of the Society was held, in October last, at Lexington, but although great preparations had been made by the physicians of that city for the reception of the Society, and though the committees appointed to make reports had been industrious in preparing for their duties, the attendance was small. The meeting was a spirited one, and the reports read were elaborate and thorough; but the state of the Treasury, owing to the small number of members present, is such that the Proceedings are not likely to be published. It is feared, that the first volume which elicited so much praise and was regarded by the Society with so just a pride, will also be the last. This is not worthy of the profession of Kentucky. We had expected a different result. The beginning was so spirited, so full of promise, that we had counted on seeing the Society go on from year to year growing in fame and usefulness, extending its researches, developing the medical history of the Commonwealth, and animating the members of our profession with a livelier zeal for its advancement. We still hope that new energy will be infused into it, and that we shall yet see it successfully pursuing its high mission. From the following card it will be seen, that Dr. R. J. Breckinridge is collecting the materials for a report on the "Medical Biography of Kentucky," and we would urge our Kentucky readers to respond to his call:

"It is the duty of the undersigned to make a report to the State Medical Society, at its next annual meeting, on the Medical Biography of Kentucky. The suitable discharge of this duty requires the assistance of medical men throughout the State. The undersigned earnestly desires to receive from *all* persons friendly to an attempt to preserve a record of this kind, brief notices or fuller sketches of deceased physicians who have spent the whole or any large part of their active professional life in this State—who have been identified with the profession here, either as teachers or practitioners of medicine.

That this record may be as complete as possible, it is desirable that the notices sent should be full and minute, though meagre ones will be more acceptable than none.

They should be forwarded before the 1st of September, and as much earlier as may be convenient.

R. J. BRECKINRIDGE.

Louisville, February, 1854.

